# English “Good Taste”

**Published:** 1872-07

**Authors:** 


### ENGLISH "GOOD TASTE."

The Doctor, an English medical monthly, which should be read by American physicians, is likely to provoke the critical anathemas of the tasteful editor of the Richmond & Louisville Medical Journal. The Doctor, in speaking of its circulation, etc., remarks:

"It may be remembered that in a Supplement to our December number, we had the satisfaction of showing that the circulation of the Doctor during the year 1871 had equalled that of any of the medical weeklies. We have now the pleasure of stating that every new number has brought us new subscribers. We are, therefore, making steady progress, and again appeal confidently for support.

The Doctor has been mentioned with approval, and its articles have been quoted, by the leading journals of Europe and America.

The Doctor has been described as—

"The pith of the periodicals at home and abroad."

"The best epitome of medical literature extant."

"The public opinion of the Medical Profession."

"A boon to the busy practitioner, as it contains in a few well-arranged pages, all that is necessary to keep any one *au courant* with the progress of medical science and literature."

"A complete review of medical practice and literature in all countries."

THE COMPANION has done no more than this—yet it has been declared to have committed "a breach of good taste, decorum, ethics and propriety." Our English cotemporary would do well to visit America and learn a lesson or two in "good taste, propriety," etc. The "good taste," etc., etc., of England is thus called in question. Of course—as Martin Chuzzlewit has it—"one of the most remarkable men in this country" is competent authority. Let England blush—and then hide her head!!

